# AI-based selection of individuals for supplemental MRI in population-based breast cancer screening: the randomized ScreenTrustMRI trial

**DOI:** 10.1038/s41591-024-03093-5

**Published:** 2024-07-08

**Authors:** Mattie Salim, Yue Liu, Moein Sorkhei, Dimitra Ntoula, Theodoros Foukakis, Irma Fredriksson, Yanlu Wang, Martin Eklund, Hossein Azizpour, Kevin Smith, Fredrik Strand

**Affiliations:** 1 Department of Oncology-Pathology, Karolinska Institutet, Stockholm, Sweden; 2 Breast Radiology Unit, Karolinska University Hospital, Stockholm, Sweden; 3 School of Computer Science and Technology, Royal Institute of Technology (KTH), Stockholm, Sweden; 4 Science for Life Laboratory, Stockholm, Sweden; 5 Department of Molecular Medicine and Surgery, Karolinska Institutet, Stockholm, Sweden; 6 Department of Medical Epidemiology and Biostatistics, Karolinska Institutet, Stockholm, Sweden; 7 Division of Robotics, Perception, and Learning, Karolinska Institutet, Stockholm, Sweden

**Keywords:** Breast cancer, Radiography, Translational research

## Abstract

Screening mammography reduces breast cancer mortality, but studies analyzing interval cancers diagnosed after negative screens have shown that many cancers are missed. Supplemental screening using magnetic resonance imaging (MRI) can reduce the number of missed cancers. However, as qualified MRI staff are lacking, the equipment is expensive to purchase and cost-effectiveness for screening may not be convincing, the utilization of MRI is currently limited. An effective method for triaging individuals to supplemental MRI screening is therefore needed. We conducted a randomized clinical trial, ScreenTrustMRI, using a recently developed artificial intelligence (AI) tool to score each mammogram. We offered trial participation to individuals with a negative screening mammogram and a high AI score (top 6.9%). Upon agreeing to participate, individuals were assigned randomly to one of two groups: those receiving supplemental MRI and those not receiving MRI. The primary endpoint of ScreenTrustMRI is advanced breast cancer defined as either interval cancer, invasive component larger than 15 mm or lymph node positive cancer, based on a 27-month follow-up time from the initial screening. Secondary endpoints, prespecified in the study protocol to be reported before the primary outcome, include cancer detected by supplemental MRI, which is the focus of the current paper. Compared with traditional breast density measures used in a previous clinical trial, the current AI method was nearly four times more efficient in terms of cancers detected per 1,000 MRI examinations (64 versus 16.5). Most additional cancers detected were invasive and several were multifocal, suggesting that their detection was timely. Altogether, our results show that using an AI-based score to select a small proportion (6.9%) of individuals for supplemental MRI after negative mammography detects many missed cancers, making the cost per cancer detected comparable with screening mammography. ClinicalTrials.gov registration: NCT04832594.

## Abstract (web summary)

In an interim analysis, an artificial intelligence model was nearly four times more efficient in terms of cancers detected per number of magnetic resonance imaging tests, compared to traditional breast density measures used in a previous clinical trial.

## Main

Mammography is the standard method for population-wide breast cancer screening, and randomized trials have confirmed that early cancer detection achieved through screening mammography leads to decreased breast cancer mortality^1,2^. However, around 30 percent of cancers in screened women are diagnosed as so-called interval cancers, becoming symptomatic after a negative screening and before the next planned screening^3^. Interval cancers may escape screening as they may be fast-growing and not present on mammograms at the time of screening, were missed by the radiologist reader or were undetectable by mammography at the time^4,5^. Like other symptomatically detected cancers, interval cancers show aggressive biology and have relatively poor prognosis^6,7^.

Contrast-enhanced MRI has been shown to be a more sensitive method for early detection of breast cancer compared with mammography^8^. The sensitivity of mammography is reduced for women with extremely dense breasts, while the sensitivity of MRI is unaffected^9–11^. In addition, women with dense breast tissue experience up to a twofold increase in breast cancer risk compared with the average woman^12–17^. The randomized DENSE trial conducted in the Netherlands demonstrated a 50% reduction in interval cancer for women invited to MRI compared with those not invited, and more than 80% reduction for women actually undergoing MRI compared with those not invited^11^. Based on those results, the cost-effectiveness ratio of offering MRI every 3 years to women with extremely dense breasts (around 8–10% of the population) has been estimated at €37,181 per QALY^18^. Due to the relatively high cost, MRI has not yet been included in any national screening program despite the European Society of Breast Imaging (EUSOBI) 2022 recommendation that women aged 50–70 years with extremely high mammographic density should have supplemental imaging by MRI or, if MRI is not available, by other methods, at least every 4 years (preferably every 2–3 years)^19^.

Previous studies have shown that reductions in the number of interval cancers can be achieved by using MRI for supplemental screening of women with high mammographic density^11,20^. The increased detection, however, comes at a substantially increased per examination cost, which results in lower cost-effectiveness compared with mammography and hinders widespread implementation^21,22^. Here, we report interim results of a prospective clinical study using a new AI image tool to select high-risk women for supplementary MRI.

The ScreenTrustMRI trial (NCT04832594) is a randomized controlled trial to assess the use of an AI tool—AISmartDensity—to select individuals for supplemental MRI. AISmartDensity was developed by the authors (Y.L., M. Sorkhei and K.S.) using data collected by authors M. Salim. and F.S. The AI tool has a modular structure with three component models assessing underlying risk, potential masking and suspicious cancer signs (Supplementary Information). The primary use case for the AISmartDensity algorithm is to triage women for supplemental MRI after negative screening mammography. The AI tool was evaluated in a retrospective clinical study where it showed a cancer detection rate up to 29 cancers per 1,000 examinations in an 8% selection of the population^23^. The primary endpoint of the ScreenTrustMRI trial is to examine the incidence of advanced cancer at 27-month follow-up after the initial screening in the group of individuals randomized to MRI compared with those randomized to no MRI. Advanced cancer, termed ‘delayed’ in the study protocol, was defined as any of the following: interval cancer, invasive component larger than 15 mm and lymph node positive cancer (that is, N1), or screen-detected cancers meeting specific criteria such as interval cancers, cancers having an invasive component larger than 15 mm or lymph node metastasis. Secondary endpoints include cancer detection at supplemental MRI, participant engagement, AI score distribution, tumor characteristics, radiological process measures and questionnaire responses. The trial started inclusions on 1 April 2021 and ended inclusions on 7 April 2023, and the follow-up period will end in August of 2025 for the last included patient. The current report focuses on the detection of additional cancers for individuals randomized to, and undergoing, supplemental MRI. It was prespecified in the study protocol that secondary endpoints could be reported before primary endpoints.

Our hypothesis was that AI-based image analysis will provide a more effective selection tool than traditional density in terms of the proportion of MRI examinations leading to a cancer diagnosis.

## Results

### Study population

**Fig. 1:**
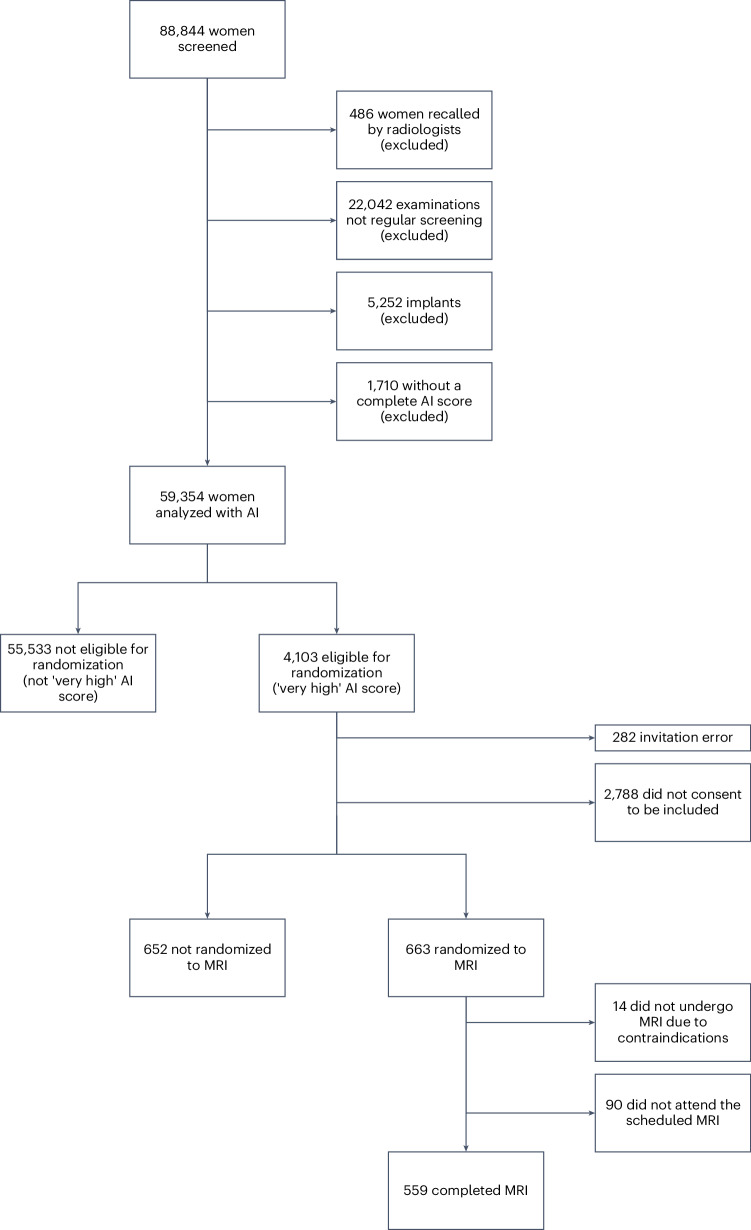
Study population. CONSORT diagram for the study population.

The source screening population for the ScreenTrustMRI trial consisted of 59,354 women whose mammograms were screened with AISmartDensity, of whom 4,103 were eligible to participate due to a ‘very high’ (top 6.9%) AISmartDensity score. The distribution of BI-RADS (Breast Imaging-Reporting and Data System) breast density^24^ and AISmartDensity assessments are shown in Supplementary Table 1. Due to an invitation error caused by delayed image processing, 282 women did not receive invitations, resulting in an invited population of 3,821 (Fig. 1).

Out of 1,315 (34% of 3,821) individuals who accepted the invitation, 663 (50% of 1,315) individuals were randomized to undergo MRI. Among those randomized to MRI, 104 (16% of 663) chose not to undergo the examination, while 559 (84% of 663) completed the MRI examination to form the final cohort for this analysis (Fig. 1). The median age of the cohort was 56 years (interquartile range (IQR) 50–65 years) (Table 1). Among participants, 22 (4% of 559) had a previous history of breast cancer and 104 (19% of 559) individuals reported having a family member with a history of breast cancer.

**Table 1:** Description of the study population of individuals undergoing MRI (*n* = 559)

All	Value
Age, years (0 missing), median (IQR)	56 (50–65)
40–49 (*n* = 129)	46 (43–47)
50–59 (*n* = 214)	54 (52–56)
60–69 (*n* = 145)	65 (63–67)
≥70 (*n* = 71)	72 (71–73)
Weight, kg (24 missing) (median, IQR)	69 (63–77)
Height, cm (13 missing) (median, IQR)	167 (163–172)
Breast self-examination (21 missing) (*n*, %)	
Never	62 (11.5%)
Occasionally	400 (74%)
Often	76 (14.1%)
Previous breast MRI (20 missing) (*n*, %)	26 (4.7%)
Previous breast cancer (13 missing) (*n*, %)	22 (4.0%)
Previous breast disease (18 missing) (*n*, %)	64 (11.8%)
Breast cancer relative (12 missing) (*n*, %)	104 (19%)
Ovarian cancer relative (14 missing) (*n*, %)	22 (4.0%)
Age at menarche (39 missing) (*n*, %)	
11 years or younger	77 (14.8%)
12–14 years	375 (72.1%)
>14 years	68 (13.1%)
Number of pregnancies (29 missing) (n, %)	
0 pregnancies	30 (5.66%)
1–2 pregnancies	244 (46.0%)
≥3 pregnancies	256 (48.3%)
Age first pregnancy (77 missing) (*n*, %)	
<19 years	46 (9.5%)
20–29 years	269 (55.8%)
>30 years	167 (34.6%)
Number of births (44 missing) (*n*, %)	
1 birth	91 (17.7%)
2–3 births	369 (71.7%)
≥4 births	19 (3.69%)
History of breastfeeding (90 missing) (n, %)	
<1 month	24 (5.1%)
1–11 months	235 (50%)
≥12 months	210 (44.8%)

Percentages do not always sum to 100% due to rounding and some values are missing due to incomplete participant reporting.

## MRI reads and subsequent procedures

A summary of the MRI results and subsequent procedures is reported in Table 2, including the radiologist assessment of each MRI examination, which was read by two breast radiologists (F.S., D.N.) with an experience level of 4 and 5 years, respectively, in MRI reading. For each MRI examination, the radiologists assessed fibroglandular proportion, corresponding to mammographic density, as well as the background parenchymal enhancement. Any lesions found were classified according to BI-RADS to assess the degree of malignancy suspicion of imaging findings: 464 (83%) were BI-RADS 1–2 (‘benign’); 54 (9.7%) were BI-RADS 3 (‘probably benign’); 27 (4.8%) were BI-RADS 4 (‘suspicious for malignancy’) and 14 (2.5%) were BI-RADS 5 (‘highly suggestive of malignancy’). In Sweden, it is common practice to biopsy BI-RADS 3 lesions and avoid short-term follow-up. Therefore, our study protocol included a first attempt at biopsy using traditional methods, but, if not possible, we applied a short-term follow-up protocol for women with BI-RADS 3 lesions. There were 95 (17%) examinations that met the condition of BI-RADS 3–5 and were flagged accordingly for further work-up. Diagnostic biopsies were obtained for 12.7% (71 of 559) of the study cohort; 71 of the 95; 62 (65%) by guided ultrasound and 9 (9.5%) by MRI. Of the 24 flagged cases who had no biopsy, 23 were BI-RADS 3 lesions that could not be localized on second-look ultrasound and 1 was BI-RADS 4 for which no corresponding lesion could be localized on second-look ultrasound or during an attempt at MRI-guided biopsy; these patients had MRI follow-up examinations scheduled at 6, 12 and 24 months per protocol; complete results for those are not yet available. In the analysis, the 24 BI-RADS 3–5 lesions that were not subjected to biopsy were classified as incomplete rather than benign, considering the unavailability of comprehensive results. Table 2 also provides an overview of the MRI assessments for individuals who were diagnosed with breast cancer and those who were not. Among the diagnosed individuals, the most common type of lesions were enhancing masses; however, among those without cancer, nonmass enhancements were more frequent.

**Table 2:** MRI radiologist reads and procedures (*n* = 559)

	Overall (*n* = 559) *n* (%)			No breast cancer (*n* = 523)^a^ *n* (%)		Diagnosed breast cancer (*n* = 36) *n* (%)		*P* value^b^
**Amount of fibrograndular tissue**								
Almost entirely fat	4	(0.7%)		4	(0.77%)	0	(0%)	N/A
Scattered fibroglandular tissue	206	(36.9%)		188	(36%)	18	(50%)	0.098
Heterogenous fibroglandular tissue	288	(52%)		275	(53%)	13	(36%)	0.057
Extreme fibroglandular tissue	59	(10.6%)		54	(10.3%)	5	(13.9%)	0.508
**Background parenchymal enhancement**								
Minimal	174	(31.3%)		161	(30.8%)	13	(36%)	0.541
Mild	230	(41.1%)		212	(40.5%)	18	(50%)	0.296
Moderate	114	(20.4%)		110	(21%)	4	(11%)	0.153
Marked	34	(6.1%)		33	(6.3%)	1	(2.8%)	0.397
**BI-RADS abnormality score**								
1 2	346 118	(61.9%) (21.1%)		346 118	(66%) (23%)	0 0	(0%) (0%)	N/A N/A
3 4 5	54 27 14	(9.7%) (4.8%) (2.5%)		47 10 2	(9%) (1.9%) (0.38%)	7 17 12	(19.4%) (47.2%) (33.3%)	0.046 <0.001 <0.001
**Type of lesion (BI-RADS** **3–5)**								
Focus	11	(1.9%)		9	(15.3%)	2	(5.5%)	0.136
Nonmass enhancement	28	(5%)		23	(39%)	5	(13.9%)	0.006
Mass	50	(8.9%)		21	(36%)	29	(81%)	<0.001

^a^Includes women that were biopsied with a benign finding and women that are undergoing follow-up.

^b^*P* value for the comparison between no breast cancer and diagnosed breast cancer.

Percentages do not always sum to 100% due to rounding. Numbers do not always equal 559 due to missing radiologist assessments. N/A, not available.

### Diagnostic outcomes

**Fig. 2:**
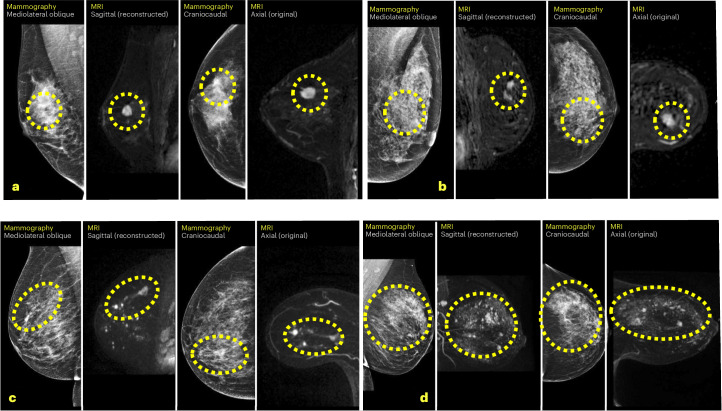
Cancers detected by supplemental MRI after negative mammography. **a**, Case A was a 13-mm large BI-RADS 4 finding on MRI that corresponded to a 13-mm large invasive cancer as diagnosed in the surgical specimen. **b**, Case B was a 9-mm large BI-RADS 4 finding on MRI that corresponded to an 8-mm invasive cancer and 14-mm ductal cancer in situ. **c**, Case C was three suspicious lesions within a total area of 60 mm, BI-RADS 5 finding on MRI, that corresponded to a 50-mm multifocal invasive lobular cancer. **d**, Case D was two suspicious lesions, the largest 13 mm, BI-RADS 3 on MRI, which corresponded to a 10-mm invasive lobular cancer with 85-mm extent including ductal cancer in situ.

Cancerous lesions were detected in 36 participants, corresponding to 64.4 (95% confidence interval (CI) 46.8–88.1) cancer detection rate per 1,000 MRI examinations (Table 3). The proportion of cancers identified in individuals recalled after MRI (positive predictive value 1 (PPV1)) was 37.9% (95% CI 28.6 to 48.1). For individuals assessed as BI-RADS 3, 4 and 5, the PPV1 was 13.0%, 63.0% and 85.7% respectively. The proportion of cancers among biopsied individuals (positive predictive value 3 (PPV3)) was 50.7% (95% CI 39.0–62.3). Figure 2 displays four cases highlighting suspicious findings on MRI, enabling comparison with the preceding negative screening mammogram. Supplementary Fig. 1 shows the results normalized to a screening cohort of 10,000 individuals with negative mammography findings and Supplementary Table 2 presents the overall AISmartDensity score and the metrics for each of the three component models across all 36 cancers.

**Table 3:** Breast cancer detection by supplemental MRI

BI-RADS	Examinations, *n*	Breast cancer detected				No breast cancer detected		
		Cancer detection rate		PPV of BI-RADS 3–5 (%)		Not recalled	Benign biopsy	Incomplete MRI follow-up
		*n*	per 1,000 MRI exams	PPV1 (95% CI)	PPV3 (95% CI)			
All	559	36	64.4 (95% CI 46.8–88.1)			464		
3–5	95	36		37.9 (28.6–48.1)	50.7 (39.0–62.3)		35	24
3	54	7		13.0 (6.2–25.1)	22.6 (10.8–41.2)		24	23
4	27	17		63.0 (42.8–79.4)	65.4 (44.7–81.5)		9	1
5	14	12		85.7 (53.5–96.9)	85.7 (53.5–96.9)		2	0

Two women decided to not pursue MRI follow-up. PPV1, PPV of BI-RADS 3–5 read; PPV3, PPV of biopsy performed after BI-RADS 3–5 read; 95% CI, estimated 95% CI by Stata ‘proportion’ command.

### Cancer characteristics

A detailed overview of the MRI and histopathological characteristics of the cancers detected is shown in Table 4. The median size of the only, or the largest, malignant lesion measured on MRI images was 12 mm (IQR 8–18 mm), which largely agreed with the histopathological analysis of the surgical specimens, which showed 13 mm (IQR 8–17). The total extent of malignancy ranged between 7 and 85 mm with a median of 15 mm (IQR 12–35). A total of 13 (36% of 36) were larger than 20 mm, corresponding to stage 2 or higher. Among all the diagnosed cancers, 7 (19% of 36) presented with multiple mass lesions on MRI whilst histopathological analysis confirmed multifocality for 4 (11% of 36). Only 3 (8% of 36) cases had lymph node metastases. In the histopathological analysis of the surgical specimens, most (22 or 61%) were a combination of invasive and ductal cancer in situ, with 5 (14% of 36) being in situ only. In terms of histological origin, according to the traditional classification system, 27 (75% of 36) cancers were classified as ductal breast cancer in the surgical specimens.

**Table 4:** Characteristics of cancers detected by supplemental MRI (*n* = 36)

	Median (IQR) or *n* (%)
**MRI**	
Lesion diameter, mm, median (IQR)	12 (8–18)
Multiple lesions, *n* (%)	
No	29 (81%)
2 lesions	5 (14%)
≥3 lesions	2 (6%)
**Biopsy** (*n* = 36)	
Invasiveness, *n* (%)	
In situ only	4 (11.1%)
Invasive only	27 (75%)
In situ and invasive	5 (13.9%)
Histology, *n* (%)	
Ductal	30 (83.3%)
Lobular	5 (13.9%)
Mucinous	1 (2.8%)
Grade, *n* (%) (1 missing)	
1	6 (17%)
2	26 (74.3%)
3	3 (8.6%)
Receptor expression, *n* (%)	
HR^+^	33 (91.7%)
HER2^+^/HR^−^	0 (0%)
HER2^−^/HR^−^	3 (8.3%)
Ki-67, *n* (%) (5 missing)	
Low	5 (14%)
Intermediate	22 (61%)
High	4 (11%)
Lymph node, *n* (%)	
No biopsy	31 (86%)
Metastasis	2 (6%)
No metastasis	3 (8%)
**Surgical specimen** (*n* = 35; 1 in neoadjuvant therapy)	
Lesion diameter, mm, median (IQR)	13 (8–17)
Total extent, mm, median (IQR)	15 (12–35)
Total extent, mm, min and max	7 and 85
Multiple lesions, *n* (%)	
No	32 (89%)
2 lesions	2 (6%)
≥3 lesions	2 (6%)
Invasiveness, *n* (%)	
In situ only	5 (13.8%)
In situ and invasive	22 (61%)
Invasive only	8 (22.2%)
Histology, *n* (%)	
Ductal	27 (75%)
Lobular	6 (16.7%)
Mucinous	1 (2.78%)
Grade, *n* (%)	
1	3 (8.3%)
2	27 (75%)
3	5 (13.9%)
Receptor expression, *n* (%)	
HR^+^	33 (91.7%)
HER2^+^/HR^−^	0 (0%)
HER2^−^/HR^−^	2 (5.6%)
Ki-67, *n* (%)	
Low	10 (27.8%)
Intermediate	14 (38.9%)
High	6 (16.7%)
Lymph node, *n* (%)	
None excised	14 (38.9%)
Metastasis	3 (8.3%)
No metastasis	19 (52.8%)

## Discussion

In this report from the ScreenTrustMRI trial, we assessed the impact of applying the AISmartDensity tool to identify women at risk of undetected cancer following negative mammography screening, focusing on those who could potentially benefit from supplemental MRI. We observed a cancer detection rate of 64.4 cancers per 1,000 MRI examinations, and a positive predictive value of 38% for individuals recalled after MRI and 50.7% for individuals who were biopsied. Most of these malignant lesions had invasive components. The invasive cancers had a median size of 13 mm on pathology analysis, which is smaller than the average size of 15.8 mm and 19.6 mm for mammography screen-detected cancer and interval cancer, respectively, previously reported for a similar breast center in the Stockholm area^25^. Using the AISmartDensity method would make the detection cost per cancer similar to the cost in population-wide screening mammography and contribute to earlier detection of invasive cancer. The cancer detection rate of our trial at 64 (95% CI 46.8–88.1) cancers per 1,000 MRIs corresponds to about 3.8 times higher supplemental cancer detection rate compared with the traditional density method used in the DENSE trial^11^ at 16.5 cancers per 1,000 MRIs. A cost-effectiveness study based on the results from the DENSE trial estimated the cost per QALY gained for supplemental MRI every 3 years at €37,181. Our results suggest that using AISmartDensity, the cost per QALY would probably be markedly lower given the close to four times higher supplemental cancer detection rate. As a alternative approach with potential to reduce MRI utilization and further increase cost-effectiveness, it may be suggested that radiologists should re-review the mammograms of all women with a very high AISmartDensity score. An assessment of this approach is planned as a post hoc reader study.

A recent meta-analysis^8^ evaluated various supplementary screening methods in women with dense breasts and a negative screening mammogram across 22 studies and a total of 132,166 individuals. Of the 22 studies, 3 focused on supplementary screening with MRI^11,26,27^, totaling 43,577 individuals screened, of whom 7,021 had dense breasts and were eligible for supplemental MRI. The pooled estimate of cancer detection rate for MRI was 25.7 per 1,000 MRI exams, with PPV1 27.7% and PPV3 34.1%. MRI was superior in terms of cancer detection compared with other supplemental screening methods: digital breast tomosynthesis, handheld ultrasound and automated breast ultrasound. One of the evaluated trials was the DENSE randomized trial, in which Bakker et al. assessed the effectiveness of supplemental MRI screening for individuals aged 50–75 years selected by traditional mammographic density^11^. Breast density was measured using automated software, and individuals with a BI-RADS D density score (corresponding to dense breast tissue) were invited to the study. The results showed that early detection by supplemental MRI screening led to a decrease in interval cancers by more than 80 percent. In the DENSE trial, the cancer detection rate among women undergoing MRI was 16.5 per 1,000 MRI examinations with a PPV for recall of 17.4%. The second trial^26^ included females aged 40–70 years of average risk (lifetime risk of 6–12%) assessed using the Gail model that uses personal medical and reproductive history, as well as the breast cancer history among first-degree relatives, but does not include breast density as a risk factor^28^. The supplementary cancer detection rate in the study was 22.6 per 1,000 examinations. The third study^27^ compared abbreviated MRI protocols (AP) with full diagnostic protocols (FP). The study included females aged 30–71 years with dense breasts (per BI-RADS) and normal mammography results, although the indication for supplementary examination in this study was unclear to us. The cancer detection rate was between 31 for AP and 33 for FP per 1,000 MRI examinations, while the PPV for recall varied between 27.8% (AP) and 41% (FP). It is notable that the additional cancer detection rate in the present study was two to four times higher than the three studies included in the meta-analysis. Traditional mammographic density and risk models thus seem to capture a markedly lower amount of relevant image information compared with the AISmartDensity tool.

When comparing mammographic density with AISmartDensity in the source population, we note associations between different mammographic density categories and the ‘very high’ category of AISmartDensity. Across all mammographic density categories, most individuals were classified as not ‘very high.’ However, within each density category there were notable variations in the proportions classified as ‘very high.’ Individuals categorized as BI-RADS D had the highest proportion 12% (682 of 5,515) classified as ‘very high’, followed by BI-RADS C 11% (3055 of 27742) and BI-RADS B 1.9% (358 of 19170). In contrast, those with BI-RADS A had the lowest proportion 0.1% (8 of 6927) classified as ‘very high.’ These findings indicate that there is some association between traditional mammographic density and AISmartDensity but far from full agreement.

Not all women with a very high density (BI-RADS D) by traditional measures are included in the very high AISmartDensity category. An important purpose of density classification is to ensure that we efficiently allocated supplemental screening resources to find as many undetected cancers as possible given a specific budget or cost-effectiveness goal. In that capacity, our results suggest that using AISmartDensity is superior to traditional density. It should be noted that, even if AISmartDensity is likely to catch around four times as many cancers as using BI-RADS D, there could be some cancers identified by BI-RADS D that AISmartDensity ‘very high’ might miss.

The results in terms of cancers detected should be put in perspective of what would be the expected future cancers without our intervention. Once the 27-month follow-up time is complete in our trial, we can compare with the women randomized to not have MRI. At present, an approximation is still possible. There were 559 individuals having MRI, which forms 6.9% of a total population of 8,101 individuals. The expected number of interval cancers in the underlying population would be 16 at the approximate 0.2% interval cancer rate in Sweden, and the expected number of next-round screen-detected cancers would be 41 at the approximate 0.5% cancer detection rate in the regular Swedish breast cancer screening system. The detected 36 cancers in the selected individuals correspond to 63% of the 57 expected future cancers in the entire screening population. The potential to pre-emptively detect most cancers by offering MRI to a small proportion of individuals represents an important healthcare value proposition.

In hindsight, some of the mammograms could be seen as showing subtle suspicious findings (Fig. 2). To understand whether this would be enough for radiologists to correctly recall them, all examinations included in this trial will be assessed in the planned post hoc reader study.

For mammography, examinations classified as BI-RADS 3 should ideally have a PPV below 2%. However, the interpretation of BI-RADS 3 in MRI is not as firmly established due to the lack of knowledge regarding features that constrain the risk to the 2% threshold. The relatively high PPV of 13% in the current study indicates that the radiologists involved were not fully calibrated to the expected 2% level and underscores the challenge in classifying BI-RADS 3 for MRI.

It is notable that most of the cancers detected in the population selected for supplementary MRI exhibited invasive features. This could be attributed to a combination of the AI tool and the use of MRI. It is known that MRI has increased sensitivity for invasive breast cancer, due primarily to increased contrast enhancement^29^, compared with more indolent cancer in situ^30^.

Lymph node metastases were relatively rare in our study. Only 8% of the 36 cases demonstrated lymph node metastasis, with 6% of cases showing evidence of this during the initial biopsy and 8% in the surgical specimen. These findings may be indicative of early detection when using MRI as a supplemental screening method. The larger tumor size observed in some of the lesions may be partially attributed to the prevalent screening nature of this study. We anticipate that the frequency of larger tumors will diminish in subsequent screening rounds. As per our study protocol, after 27 months follow-up we will report differences between the MRI arm and the control arm in terms of the number of cancers with adverse prognostic features.

Based on results from the previously published retrospective evaluation, it would seem that the cancer signs part of the AISmartDensity score would be just as good, in terms of AUC, as the summary score. However, in the current prospective implementation, 16 of 36 cancers actually had a higher masking or risk score than cancer signs score (Supplementary Table 2)^23^. The ground truth on which the AUC calculation is based naturally differ between the retrospective evaluation (based on a 3-year follow-up time) and the prospective implementation (cancers that near time of screening are actually detected by MRI). We believe that the three components adds robustness and are valuable in practice.

The main strength of this study is its prospective nature, using a population derived from a well-controlled prespecified randomized clinical trial conducted within a population-based screening program. The use of the automated AISmartDensity assessment ensured there was no interreader variability in mammogram assessment. The software was developed in-house (patent pending) and has not yet received CE mark or United States Food and Drug Administration approval. A key limitation of the present report is that the cancer detection rate using this method can be compared only with results using traditional density in other studies, not within this study. Also, a limitation is that we do not have patient parameters required to compare with traditional risk model scores. Furthermore, although this study provides clear proof-of-principle, the AI tool was trained only on mammography images from Hologic equipment, using images of high quality assessed by highly experienced radiologists will need to be validated for other equipment for the method to be generalized. Additionally, the trial did not explicitly assess the efficacy of AISmartDensity in women with a history of cancer. The small number of cancer cases also hinders the possibility of conducting subgroup analyses related to cancer characteristics and the incomplete results from MRI follow-up of the 24 BI-RADS 3–5 lesions have the potential to bias results towards more conservative estimates. Furthermore, it should be noted that the applicability of this study may be limited to centers or regions with similar breast cancer screening programs and intervals. Additionally, the women who chose to participate in the study may have a higher incidence of cancer compared with the average population, for example, caused by being more aware of the risk of cancer due to having a higher frequency of cancer in their family history^31,32^. However, women with a strong family history of breast cancer or genetic mutation are all excluded, as are women who have already noticed a lump in their breast. It is important to clarify that our study exclusively reports on the prevalence round of supplemental MRI screening, that is, the first time this approach is used. For our study, as for any prevalence screening, one would expect a higher cancer detection rate compared with previous and subsequent screening rounds. We would also expect that the stage distribution of screen-detected cancers is shifter towards lower stages compared with previous mammography-only rounds and will be further shifted towards lower stages in subsequent rounds.

In conclusion, using an approach of analyzing mammograms with AI as a selection method for supplemental MRI is around four times more efficient, in terms of cancer detection, than second-best approaches based on traditional density measures and risk models. Subsequent follow-up research will determine primary outcomes of the effect on prognostically important cancer characteristics.

## Methods

The ScreenTrustMRI trial (ClinicalTrials.gov: NCT04832594) is a randomized trial to evaluate the use of AI to select individuals for supplemental MRI within population-based breast cancer screening at Karolinska University Hospital. Participants in the study were enrolled between 1 April 2021, and 7 April 2023. The current report focuses on predefined secondary outcomes, and an ad hoc cost-comparison analysis, based on a subset of the study population as discussed below.

### Study setting

The study was conducted at the breast imaging department of Karolinska University Hospital. Under the Swedish national breast screening program, women between 40 and 74 years old are invited for mammographic screening every 2 years. After the age of 74 years, individuals may sometimes undergo screening on their own request. Screening consists of a two-view full-field digital mammogram of each breast. At Karolinska University Hospital, the screening examination is assessed with nonblinded double-reading (that is, the second reader can view the decision by the first reader). Examinations flagged by either reader are routed to a consensus discussion between two radiologists, who decide whether to recall a woman or not. There are no other methods than mammography for the general population, that is, no tomosynthesis, ultrasound or MRI, and radiologists do not report on mammographic density. All mammograms in the study were obtained on Hologic 3Dimensions mammography equipment.

### AISmartDensity—software analyzing mammograms

All screening mammograms at Karolinska University Hospital were analyzed by the AISmartDensity model, which is image analysis software developed collaboratively by investigators at the Karolinska Institute and at the Royal Institute of Technology in Stockholm, Sweden^23^. Briefly, it comprises three types of AI models that utilize previously well-described convolutional neural network approaches for image classification: (1) inherent breast cancer risk—an EfficientNetB3 architecture trained on images of females without current cancer, with the task being to classify females likely to be diagnosed with breast cancer within the next 5 years from those remaining healthy; (2) masking potential—a ResNet34 architecture trained to rank normal mammograms by varying difficulty levels based on radiologists’ subjective assessments; and (3) cancer signs—a ResNet34 architecture trained to classify between images with current cancer and those with normal mammograms. AISmartDensity uses an average of standardized scores from the three models. For the cancer signs model, the in-house model was combined with the output of a similar commercial AI (Insight MMG, Lunit Inc.), which also generated conventional breast density measures. The training data for the in-house algorithms were derived mainly from the CSAW dataset, obtained from the Karolinska University Hospital^33^. AISmartDensity is calculated as the age-adjusted sum of the standardized scores from each model. The ‘very high’ category of AISmartDensity, was calibrated using the retrospective development data to align with the top 8% of the AISmartDensity score, corresponding to a cut-off value of 1.97 (ref. ^23^).

### Eligibility criteria and enrollment

Women who participated in the public screening program who had a standard four-view mammography examination and a ‘very high’ AISmartDensity score of their screening mammogram were eligible for the trial. Individuals attending screening as part of a high-risk surveillance program (for example, personal history, family history or genetic mutations) undergo supplemental imaging and were therefore excluded from the trial. Additionally, individuals with breast implants, individuals recalled for diagnostic work-up after mammography, and individuals whose images could not be processed by the AI tool were also excluded. Eligible women received an invitation letter and written trial information. Those providing written informed consent were enrolled and randomized either to supplemental breast MRI or as part of an observational control arm, which is not included in the current analysis. Randomization was performed by having a random number generated in Microsoft Excel by a study administrator not involved in the radiological assessments. The randomization procedure did not include stratification or blocks. Participants randomized to the MRI group received an appointment for the examination within 3 months of their initial screening, with a few exceptions. Each participant randomized to MRI only had one screening MRI. For the current report, only individuals who underwent MRI are included in this analysis.

Women randomized to supplementary MRI were sent a questionnaire on breast cancer risk factors and history, as well as potential contraindications to MRI. Individuals who had absolute or relative contraindications against MRI, including those breastfeeding or pregnant, were excluded from having MRI.

### MRI protocol

All MRI images were obtained on a GE Signa Premier 3 T MRI scanner. We composed a dedicated MRI protocol for the trial with a total scan time of 12 min. The protocol consists of a localizer, a 2D Axial Fast Spin-Echo T2-weighted Dixon imaging sequence (FA = 111, TE = 102 ms and TR = 5,365 ms, 1.1 × 1.1-mm matrix size and 4-mm slice thickness), a T1-weighted dynamic contrast-enhanced Dixon sequence (FOV = 35 mm, 0.9 × 0.9-mm in-plane and 1.4-mm slice thickness) with view-sharing enabled to achieve desired times. The dynamic contrast-enhanced sequence contained seven timeframes acquired at 0:00, 0:33, 1:04, 1:37, 3:00, 4:30 and 6:00 min after contrast injection. Based on the subtraction images from the fourth time frame, a maximum intensity projection image was produced.

### MRI interpretation and diagnostic workflow

MRI images were assessed by two nonblinded breast radiologists and classified according to the ACR MRI BI-RADS classification. For each MRI examination the radiologists assessed fibroglandular proportion (from ‘A. almost entirely fat’ to ‘D. extreme fibroglandular tissue’), corresponding to mammographic density, as well as the background parenchymal enhancement. Any lesions found were classified according to malignancy suspicion of imaging findings (1–5). For examinations with more than one lesion, the BI-RADS score on exam-level was defined by the highest score for any individual lesion. The second reader was instructed to seek consensus by discussing with the first reader for any marked disagreement. Examinations classified as BI-RADS 1 or 2 (negative, benign) were considered healthy and did not undergo further assessment. Examinations with a finding classified as BI-RADS 3, 4 or 5 (probably benign, suspicious, highly suggestive of malignancy) were recalled for a second-look ultrasound. If a lesion could be identified, it was biopsied to obtain a pathology-verified diagnosis. For BI-RADS 3 lesions not visualized on ultrasound, follow-up MRIs at 6, 12 and 24 months were assigned. For BI-RADS 4–5 lesions not visualized on ultrasound, MRI-guided biopsies were arranged. The presence or absence of cancer was confirmed through histopathology. Cases with BI-RADS 4–5 findings were reported to the multidisciplinary team conference and the patient responsibility transferred to the breast surgery department.

### Outcomes

Biopsy-verified breast cancer detected by MRI after a negative screening mammography was the primary outcome measure in this report. Breast cancer was defined as any invasive cancer in the breast or ductal cancer in situ. As permitted by the informed consent, we follow each patient for 27 months after diagnosis. For this report, we extracted pathology assessments for the diagnostic biopsy and for the surgical specimen. Also, we recorded the radiologist interpretations of the MRI examinations and subsequent diagnostic work-up. All study participants will have a subsequent follow-up period of 27 months to assess the total number of cancers with adverse prognostic characteristics, that is, interval cancer, larger than 2 cm invasive cancer, lymph node positive cancer.

### Cost-comparison

A high-level cost-comparison analysis was carried out for a range of cancer detection rates from 0 to 120 per 1,000 examinations. An important assumption was that MRI is ten times more expensive per examination compared with mammography. We plotted a curve describing the relative cost per detected cancer compared with mammography. The cancer detection rate, and corresponding relative cost, observed in our trial and that observed in a previous trial using traditional density as the selection mechanism were determined.

### Statistical analysis

Cancer detection rate was expressed as number of cancers detected per 1,000 patients receiving MRI, calculated by dividing the number of individuals with pathology-verified breast cancer by the number completing MRI screening, multiplied by 1,000. For PPV we used two metrics. The first metric (PPV1) was based on dividing the number of individuals with pathology-verified cancer by the number with a positive MRI screening result (BI-RADS 3–5). The second metric (PPV3) was based on the denominator of all individuals that underwent biopsy. Stata statistical software v.15.1 was used for all statistical analyses. All statistical tests were two-sided. The level for statistical significance was set at alpha = 0.05. There was no adjustment for multiple testing.

### Study oversight

The trial was approved by the ethics review board in Stockholm County and was monitored by an independent Data Safety and Monitoring Committee. The authors assume responsibility for the accuracy and completeness of the data and analyses, and for the adherence of the trial to the study protocol. The study complies with all local and national regulations regarding the use of human study participants and was conducted in accordance to the criteria set by the Declaration of Helsinki. The authors of this manuscript vouch for the accuracy and completeness of the data reported.

### Reporting summary

Further information on research design is available in the Nature Portfolio Reporting Summary linked to this article.

## Online content

Any methods, additional references, Nature Portfolio reporting summaries, source data, extended data, supplementary information, acknowledgements, peer review information; details of author contributions and competing interests; and statements of data and code availability are available at 10.1038/s41591-024-03093-5.

## Supplementary information

Supplementary Information Supplemental Tables 1 and 2 and Fig. 1.

Reporting Summary

## Data Availability

De-identified patient-level prediction scores and diagnostic outcomes will be shared upon request to the corresponding author who will respond within 4 weeks. Other patient-level data, including images, will be shared as far as allowed by applicable regulations including the GDPR and approval by the Karolinska legal department in relation to patient data integrity. An anonymized version of the CSAW dataset involved in training the in-house algorithms is available at 10.5878/45vm-t798.

## Code availability

Source code is available at https://github.com/radiology2023/RetrospectiveAISmartDensity.
