# Supplementary material for: AI-based selection of individuals for supplemental MRI in population-based breast cancer screening: the randomized ScreenTrustMRI trial
Source: Nat Med. 2024 Jul 8;30(9):2623–30. doi: 10.1038/s41591-024-03093-5 (PMC11405258; doi:10.1038/s41591-024-03093-5)
Supplement: Supplementary file 1 — Supplemental Tables 1 and 2 and Fig. 1. [file 41591_2024_3093_MOESM1_ESM.pdf]

nature medicine

Supplementary information https://doi.org/10.1038/s41591-024-03093-5

AI-based selection of individuals for supplemental MRI in population-based breast cancer screening: the randomized ScreenTrustMRI trial

In the format provided by the authors and unedited

CONTENTS

page 2: Supplemental Table S1. Mammographic density and AISmartDensity in the source screening population

page 3: Supplemental Table S2. AISmartDensity Composite Score and Component Measures for Diagnosed Cancers

page 4: Supplemental Figure 1: Results normalized to a population of 10,000 individuals where no cancer was detected by screening mammography. AISmartDensity was calculated, and the individuals with the top 6.9% of AISmartDensity scores underwent supplemental MRI.

Supplemental Table S1. Mammographic density and AISmartDensity in the source screening population (n=59,354)

Mammographic density	AISmartDensity					
	Any		'Very high'		Not 'Very high'	
	n	(%)	n	(%)	n	(%)
All	59354	(100%)	4103	(6.9%)	55251	(93%)
BIRADS A (Lowest)	6927	(12%)	8	(0.1%)	6919	(100%)
BIRADS B	19170	(32%)	358	(1.9%)	18812	(98%)
BIRADS C	27742	(47%)	3055	(11%)	24687	(89%)
BIRADS D (Highest)	5515	(9%)	682	(12%)	4833	(88%)

Mammographic density estimated by computer software Lunit INSIGHT MMG

Supplemental Table S2. AISmartDensity Composite Score and Component Measures for Diagnosed Cancers (n=36)

Cancer	AISmartDensity	Masking	Risk	Cancer Signs
1	2.2	0.5	2.1	1.0
2	2.5	1.9	0.5	0.3
3	2.8	-0.1	1.2	3.0
4	2.1	1.2	0.0	0.9
5	2.0	1.2	1.9	0.8
6	2.2	0.9	2.2	0.6
7	2.1	0.9	2.2	0.3
8	3.1	1.3	2.5	2.2
9	2.4	-0.9	1.5	3.8
10	3.3	1.0	1.1	3.9
11	4.0	1.8	1.7	3.6
12	2.8	0.1	0.1	3.9
13	2.1	0.3	2.0	1.4
14	3.1	1.1	2.2	2.0
15	2.2	1.5	0.6	0.6
16	2.0	0.8	1.6	1.0
17	2.4	-0.4	1.6	2.8
18	2.5	0.2	0.4	2.8
19	2.2	0.8	-0.6	2.0
20	2.7	0.4	0.3	2.9
21	2.3	-0.2	1.7	2.8
22	2.3	1.7	0.2	0.9
23	4.3	1.9	2.1	3.6
24	2.2	0.5	1.5	1.2
25	2.0	0.9	1.6	0.6
26	3.3	1.5	1.4	1.0
27	2.3	1.1	1.2	0.6
28	3.0	0.2	0.6	3.1
29	2.7	0.3	1.5	3.2
30	3.9	0.8	0.6	3.9
31	3.0	0.6	1.2	3.1
32	2.6	-0.5	1.0	3.9
33	2.0	1.1	1.3	1.1
34	2.4	1.1	0.6	2.2
35	3.7	1.4	-1.1	3.6
36	3.2	1.8	1.7	1.3

*Note: The numbers presented in the table are standardized. The summary score AI Smart Density was calculated as Masking + Risk + Cancer Signs * (110 - patient age) / 70*

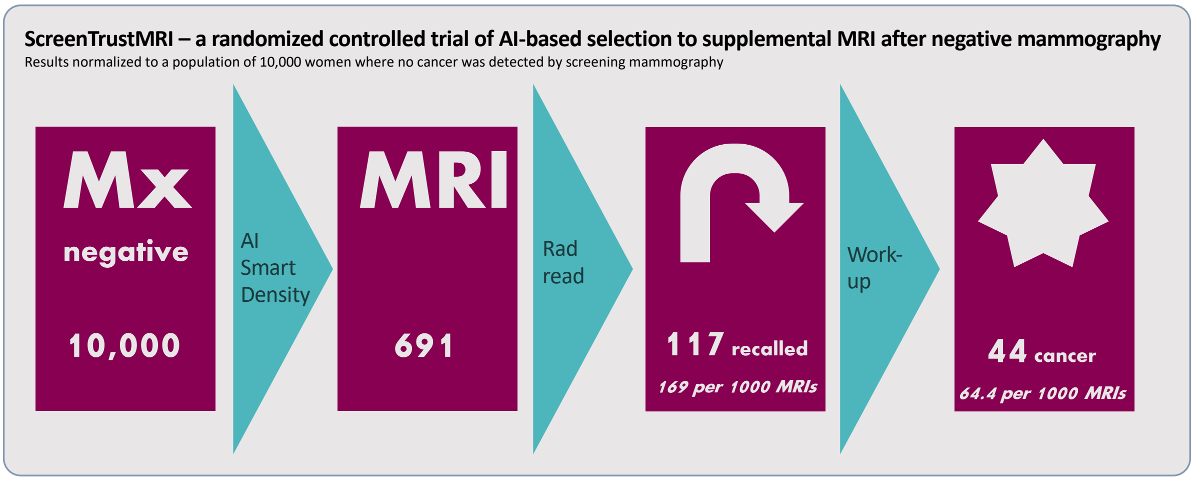

Supplemental Figure 1. Results normalized to a population of 10,000 individuals where no cancer was detected by screening mammography. AISmartDensity was calculated, and the individuals with the top 6.9% of AISmartDensity scores underwent supplemental MRI.
