# Supplementary material for: AI-based selection of individuals for supplemental MRI in population-based breast cancer screening: the randomized ScreenTrustMRI trial
Source: Nat Med. 2024 Jul 8;30(9):2623–30. doi: 10.1038/s41591-024-03093-5 (PMC11405258; doi:10.1038/s41591-024-03093-5)
Supplement: Supplementary file 2 — Reporting Summary [file 41591_2024_3093_MOESM2_ESM.pdf]

nature portfolio

Corresponding author(s): Fredrik Strand

Last updated by author(s): May 10, 2024

Reporting Summary

Nature Portfolio wishes to improve the reproducibility of the work that we publish. This form provides structure for consistency and transparency in reporting. For further information on Nature Portfolio policies, see our Editorial Policies and the Editorial Policy Checklist.

Statistics

For all statistical analyses, confirm that the following items are present in the figure legend, table legend, main text, or Methods section.

n/a	Confirmed	
☐	☒	The exact sample size (n) for each experimental group/condition, given as a discrete number and unit of measurement
☐	☒	A statement on whether measurements were taken from distinct samples or whether the same sample was measured repeatedly
☐	☒	The statistical test(s) used AND whether they are one- or two-sided<br>*Only common tests should be described solely by name; describe more complex techniques in the Methods section.*
☐	☒	A description of all covariates tested
☐	☒	A description of any assumptions or corrections, such as tests of normality and adjustment for multiple comparisons
☐	☒	A full description of the statistical parameters including central tendency (e.g. means) or other basic estimates (e.g. regression coefficient) AND variation (e.g. standard deviation) or associated estimates of uncertainty (e.g. confidence intervals)
☐	☒	For null hypothesis testing, the test statistic (e.g. F, t, r) with confidence intervals, effect sizes, degrees of freedom and P value noted<br>*Give P values as exact values whenever suitable.*
☒	☐	For Bayesian analysis, information on the choice of priors and Markov chain Monte Carlo settings
☒	☐	For hierarchical and complex designs, identification of the appropriate level for tests and full reporting of outcomes
☒	☐	Estimates of effect sizes (e.g. Cohen's d, Pearson's r), indicating how they were calculated

Our web collection on statistics for biologists contains articles on many of the points above.

Software and code

Policy information about availability of computer code

Data collection	Custom code, in-house, https://github.com/radiology2023/RetrospectiveAISmartDensity
Data analysis	Stata statistical software version 15.1 for all statistical analyses. Microsoft Excel version 16.72

For manuscripts utilizing custom algorithms or software that are central to the research but not yet described in published literature, software must be made available to editors and reviewers. We strongly encourage code deposition in a community repository (e.g. GitHub). See the Nature Portfolio guidelines for submitting code & software for further information.

Data

Policy information about availability of data

All manuscripts must include a data availability statement. This statement should provide the following information, where applicable:

- Accession codes, unique identifiers, or web links for publicly available datasets
- A description of any restrictions on data availability
- For clinical datasets or third party data, please ensure that the statement adheres to our policy

De-identified patient-level prediction scores and diagnostic outcomes will be shared upon request to the corresponding author who shall respond within four weeks. Other patient-level data including images, will be shared as far as allowed by applicable regulations including the GDPR and approval by the Karolinska legal department in relation to patient data integrity. An anonymized version of the CSAW dataset involved in training the in-house algorithms is available at: https://doi.org/10.5878/45vm-t798

Research involving human participants, their data, or biological material

Policy information about studies with human participants or human data. See also policy information about sex, gender (identity/presentation), and sexual orientation and race, ethnicity and racism.

Reporting on sex and gender	Mammography screening participants, female-only
Reporting on race, ethnicity, or other socially relevant groupings	N/A
Population characteristics	Age IQR (50-65), Weight IQR (63-67), Height IQR (163-172), Previous breast cancer 4%, Previous breast disease 11.8%, surgery 35/36.
Recruitment	All women were invited by letter after completed negative screening mammogrpahy. Upon agreeing to participate we used random sampling to minimize self-selection bias and ensure the generalizability of our findings to the target population.
Ethics oversight	The trial was approved by the ethics review board in Stockholm County and was monitored by an independent Data Safety and Monitoring Committee.

Note that full information on the approval of the study protocol must also be provided in the manuscript.

Field-specific reporting

Please select the one below that is the best fit for your research. If you are not sure, read the appropriate sections before making your selection.

☒ Life sciences ☐ Behavioural & social sciences ☐ Ecological, evolutionary & environmental sciences

For a reference copy of the document with all sections, see nature.com/documents/nr-reporting-summary-flat.pdf

Life sciences study design

All studies must disclose on these points even when the disclosure is negative.

Sample size	The sample size calculation relates to the primary analysis after women have undergone the baseline mammogram. We have estimated that the study would have a at least 90% power in detection of a statistically significant ($p<0.05$) difference in the absolute incidence of the primary endpoint of significantly delayed cancer
Data exclusions	5,252 exams with breast implants; 1,710 exams without complete AI score. The exclusions were pre-specified.
Replication	Code sharing
Randomization	Randomization was performed by having a random number generated in Microsoft Excel by a study administrator not involved in the radiological assessments. The randomization procedure did not include stratification or blocks. Participants randomized to the MRI group received an appointment for the examination within three months of their initial screening, with a few exceptions. For the current report, only individuals who underwent MRI are included in this analysis.
Blinding	Blinding was not applicable to our study due to the nature of the study design. However, we took steps to minimize bias through randomization.

Reporting for specific materials, systems and methods

We require information from authors about some types of materials, experimental systems and methods used in many studies. Here, indicate whether each material, system or method listed is relevant to your study. If you are not sure if a list item applies to your research, read the appropriate section before selecting a response.

Materials & experimental systems

n/a	Involved in the study
☒	☐ Antibodies
☒	☐ Eukaryotic cell lines
☒	☐ Palaeontology and archaeology
☒	☐ Animals and other organisms
☐	☒ Clinical data
☒	☐ Dual use research of concern
☒	☐ Plants

Methods

n/a	Involved in the study
☒	☐ ChIP-seq
☒	☐ Flow cytometry
☒	☐ MRI-based neuroimaging

Clinical data

Policy information about clinical studies

All manuscripts should comply with the ICMJE guidelines for publication of clinical research and a completed CONSORT checklist must be included with all submissions.

Clinical trial registration

NCT04832594

Study protocol

https://classic.clinicaltrials.gov/ProvidedDocs/94/NCT04832594/Prot_SAP_001.pdf

Data collection

From the Karolinska University Hospital. The trial started inclusions on 1st April 2021 and ended inclusions on the 7th April 2023.

Outcomes

Primary Outcome(s)
The primary outcome is a composite endpoint representing early detection failure – a breast cancer diagnosed with any of the below characteristics:
- Interval cancer
- Cancer with invasive component larger than 15 mm
- Cancer with lymph node metastasis

The follow-up time starts once the initial mammography and screening MRI are fully processed and any cancer detected at the initial examination has been diagnosed. Thus, the cancers that will be considered for primary outcome are the women that are healthy after the initial examination. A positive primary outcome will be based on interval cancers and the cancers that are screen-detected during a 27-month follow-up time after initial screening and fulfil the above criteria.
Secondary Outcome(s)
To the extent that a secondary outcome measure does not overlap with the primary outcome, it may be included in interim reporting.
1. Women invited, women declined and women accepted to participate; including reason for nonparticipation (contraindication, patient choice, no response).
2. Distribution of AI scores for: i) Joint model, ii) AI risk score, iii) AI masking score, iv) AI CAD score(s).
3. a) Breast cancer diagnosed by screening MRI.
b) All breast cancers diagnosed during the study time including the initial screening, including mode of detection (MRI, mammographic detection, clinical detection including time since screening) for all women for whom AI scores were calculated (above and below threshold)
For all cancers in the outcome measures, the following tumor characteristics will be reported:
a) Invasiveness (in situ or invasive; micro-invasive counted as in situ)
b) Histology (ductal, lobular, mucinous, tubular, other)
c) Lymph node status (0, 1-3, or more than 4 lymph node metastases)
d) Tumor size (in mm) – in situ and invasive component separately measured by pathologist
e) Ki-67 percent. With a 20% binary cut-off, and an ex 14% cut-off level
d) Molecular subtype (Luminal A-like defined as estrogen and/or progesterone receptor positive and HER2 negative and KI-67 <14%, Luminal B-like defined as estrogen and/or progesterone receptor positive and HER2 positive/negative and KI-67 >=14% , HER2-positive defined as estrogen and progesterone receptor negative and HER2 positive, and
8 / 24
Triple negative defined as estrogen and progesterone receptor and HER2 negative)
4. Radiological process measures: distribution of BI-RADS scores (amount of fibroglandular tissue, background parenchymal enhancement, and any lesion); number and outcome of second look ultrasound, biopsies, short-term MRI follow-up. We will report operational characteristics of the screening MRI (cancer detection rate, recall rate, positive predictive value for 2nd look ultrasound decision and for biopsy decision).
5. Questionnaire for women undergoing MRI: self-examination habit (yes, sometimes, often); previous MRI (yes, no); prior breast cancer (yes, no, which breast and year); prior non-malignant breast disease (yes, no, which year); first-degree relative with breast cancer (yes, no, at what earliest age); first-degree relative with ovarian cancer (yes, no, earliest age); Age of menarche, Parity, Age of first pregnancy, Number of children born, Time of breast-feeding, date of latest menstruation; reason for no menstruation, use of hormonal medications; use of Cozaar or Losartan; Current length and weight. They may also fill in a questionnaire concerning their experience of undergoing a screening MRI.
Explorative: The influence on cancer incidence and detection of the following potential predictors or modifiers will be explored (further detailed in the Statistical Analysis Plan): Radiologist assessments of

screening mammogram at inclusion, Age, Density, AI scores: Overall pipeline (the basis for cut-off point), and various cut-off points for each of the three AI components (risk, masking and CAD).

Plants

Seed stocks	N/A
Novel plant genotypes	N/A
Authentication	N/A
